# Frailty and Fitness in Older Adults With Acute Myeloid Leukemia: From Chronological Age to Multiparametric Assessment

**DOI:** 10.1111/ejh.70223

**Published:** 2026-05-25

**Authors:** Antonella Bruzzese, Enrica Antonia Martino, Santino Caserta, Maria Eugenia Alvaro, Nicola Amodio, Eugenio Lucia, Virginia Olivito, Caterina Labanca, Francesco Mendicino, Fortunato Morabito, Ernesto Vigna, Massimo Gentile

**Affiliations:** ^1^ Hematology Unit, Department of Onco‐Hematology AO of Cosenza Cosenza Italy; ^2^ Department of Experimental and Clinical Medicine University of Catanzaro Catanzaro Italy; ^3^ Department of Pharmacy, Health and Nutritional Science University of Calabria Rende Italy; ^4^ AIL Sezione di Cosenza Cosenza Italy

**Keywords:** acute myeloid leukemia, comorbidity index, comprehensive geriatric assessment, elderly patients, fitness assessment, frailty

## Abstract

Acute Myeloid Leukemia predominantly affects older adults, who often present with comorbidities, functional impairment, and frailty, limiting eligibility for intensive chemotherapy or allogeneic transplantation. Historically, treatment options were restricted to hypomethylating agents, low‐dose cytarabine, hydroxyurea, or supportive care, with poor outcomes. The introduction of hypomethylating agents combined with Venetoclax and the availability of targeted therapies have expanded therapeutic possibilities, challenging the notion that elderly AML is uniformly incurable. In this evolving context, accurate assessment of fitness and frailty is essential to guide treatment decisions. This review summarizes current concepts of frailty in older AML patients, including general geriatric tools and hematology‐specific indices. It discusses the role of Comprehensive Geriatric Assessment, frailty phenotypes, and comorbidity scores such as the transplantation‐specific comorbidity index and the Charlson index, alongside AML‐focused tools like the Ferrara criteria and integrated molecular risk models. Most existing tools were developed in intensively treated cohorts, and their applicability to patients receiving non‐intensive regimens remains uncertain. Fitness is increasingly recognized as a dynamic, multidimensional construct incorporating clinical, functional, cognitive, social, and biological factors. A multiparametric approach is proposed, integrating these variables into decision‐making models, potentially enhanced by artificial intelligence to optimize treatment selection in older AML patients.

## 1 Introduction

The term acute myeloid leukemia (AML) encompasses a heterogeneous group of clonal hematopoietic disorders with diverse clinical and biological features. Curative‐intent treatment has traditionally relied on intensive chemotherapy (IC), with or without consolidation using allogeneic stem cell transplantation (alloSCT), according to risk stratification [1]. Although AML can occur at any age, it predominantly affects older adults; in fact, most newly diagnosed patients are elderly and frequently considered unfit for IC and alloSCT because of advanced age, comorbidities, or frailty [2].

Historically, treatment options for these patients have shown limited efficacy and included hypomethylating agents (HMAs), both azacitidine (AZA) and decitabine (DEC), hydroxyurea, low‐dose cytarabine, or best supportive care (BSC) alone [1]. In recent years, however, advances in the understanding of AML biology have led to the development of targeted agents and novel combinations that have transformed the management of this vulnerable population, challenging the historical notion that the elderly represent an incurable group [3, 4, 5].

Concurrently, growing attention has been directed toward both the biological underpinnings of AML and the optimization of care for frail patients, with the dual aim of improving efficacy and reducing treatment‐related toxicity. The European LeukemiaNet (ELN) now recommends specific strategies for patients deemed unfit for intensive treatment and emphasizes the need for more refined fitness stratification based on a combination of patient‐ and disease‐related factors [1]. In this context, systematic assessment of fitness and comorbidities has become a crucial component of the diagnostic workup in all AML patients, as it directly informs the choice and intensity of therapy.

Comprehensive Geriatric Assessment (CGA), evaluating clinical, cognitive, physical, and social domains, has been explored as a prognostic tool in elderly AML. However, its complexity and time requirements limit routine implementation, and no consensus has been reached regarding its standardized use in daily practice. To address these limitations, several simplified tools have been proposed, some of which incorporate disease‐related variables such as cytogenetic and molecular abnormalities—for example, the AML Composite Model (AML‐CM) [6, 7, 8, 9, 10, 11, 12, 13, 14, 15, 16, 17]. Although these scores have improved the ability to classify fitness, their prognostic value in AML patients receiving non‐intensive therapy remains uncertain because of the paucity of validation studies in real‐world settings.

The objective of this review is to critically summarize current evidence on frailty and fitness assessment in older adults with AML, describing general geriatric and hematology‐specific tools, their prognostic value and limitations—particularly in the context of non‐intensive and targeted therapies—and to propose a practical, multiparametric approach that integrates patient‐related and disease‐related factors to guide treatment selection in this population.

### 1.1 Definition and Evaluation of Frailty

The term “fitness” describes a multidimensional construct that includes age, performance status (PS), comorbidities, and functional capacity. Advanced age is one of the most important determinants of fitness, as the prevalence of frailty increases with aging. Frailty manifests as an increased vulnerability to stressors and decreased physiological reserve, resulting in a limited capacity to maintain homeostasis. It is driven by multisystem physiological changes that do not necessarily cause overt disease, but contribute to a general impairment of cellular responses—such as apoptosis, senescence, and repair—and to dysregulation of immune activation and inflammation pathways [18].

It remains unclear whether age‐related frailty in the “oldest‐old” is predominantly a direct consequence of the aging process itself or is mainly driven by the cumulative effect of subclinical diseases. This uncertainty raises the question of whether age can be isolated from other determinants of frailty and considered an independent causal factor, or whether advanced age merely represents a surrogate for comorbidities and other deficits [19]. In any case, frailty confers increased susceptibility to a wide range of diseases and treatment‐related complications. The extent to which frailty influences the clinical management of older patients is still debated, and several frailty indices have been proposed over the years.

The CGA, first introduced in 1989, suggests that frailty is only partially related to advanced age and is more strongly influenced by functional capacity [20, 21]. The CGA is a multidimensional diagnostic process that evaluates functional abilities and impairments across different domains of geriatric patients. In 2012, Bellera et al. developed the G8, a screening tool designed to identify older patients with cancer who may benefit from a full CGA. The G8 consists of seven items from the Mini Nutritional Assessment (MNA) questionnaire plus age, and it is benchmarked against seven CGA scales: Activities of Daily Living (ADL), Instrumental ADL (IADL), MNA, Mini‐Mental State Exam (MMSE), Geriatric Depression Scale (GDS), Cumulative Illness Rating Scale‐Geriatrics (CIRS‐G), and Timed Get Up and Go (TGUG). The presence of at least one impaired questionnaire score is considered abnormal [22].

In 2001, Fried et al. described the “frailty phenotype” as a clinical syndrome defined by the presence of at least three of the following criteria: weakness (grip strength), unintentional weight loss, self‐reported exhaustion, slow walking speed, and low physical activity. The frail phenotype was more prevalent with increased age, and was associated with female sex, lower income and education, African American race, poorer health status, and higher rates of comorbid chronic diseases and disability. In the Cardiovascular Health Study, the prevalence of frailty was 6.9% overall, increasing from 3.2% in those aged 65–70 to 25.7% in those aged 85–89 years; by race, prevalence was 12.9% in African Americans compared to 6.5% in whites [23].

There was overlap, but not complete concordance, between frailty, comorbidity, and disability. The frailty phenotype was independently associated with increased risks of hospitalization, incident falls, worsening mobility or ADL disability, and death. Individuals with an intermediate frailty status (one or two criteria) had an intermediate risk for these outcomes and a higher likelihood of progressing to frail within 3–4 years [23].

The Vulnerable Elders Survey (VES‐13) is a multivariate model that uses functional status, self‐rated health, and age to predict death or functional decline. It yields a 13‐item function‐based score; a value ≥ 3 identified approximately one‐third of older adults as vulnerable with 4.2‐fold higher risk of death or functional decline over 2 years compared with those with lower scores. This tool has proven to be an efficient system for identifying older people at risk, relying on self‐reported information and being easily applicable across care settings. Notably, the addition of self‐reported diagnoses and conditions does not enhance its predictive ability [24].

The Clinical Frailty Scale (CFS) is a visual seven‐point tool (later expanded to nine points) that has demonstrated a high correlation with the Frailty Index. Each one‐category increment on the CFS is associated with a significant rise in medium‐term risks of death and institutionalization, even after adjustment for age, sex, and education [25].

### 1.2 Frailty in Hematological Malignancies

With global population aging and improved diagnostic capabilities, the number of older patients with hematological malignancies is steadily rising. Nevertheless, few clinical trials are specifically designed for older adults, resulting in limited data to estimate chemotherapy toxicity or tolerability in this age group. Most clinical trial participants are younger than 65 years old, and the estimated enrolment rate of older adults with blood cancers in clinical trials is only 11% [26].

In the absence of robust prospective data, clinicians often rely on age, comorbidities, and PS to estimate the risk of chemotherapy‐related toxicity in daily practice. However, no single parameter can reliably predict life expectancy, functional capacity, or the risk of treatment complications [27]. Furthermore, aging is a heterogeneous process; chronological age alone does not adequately reflect global health status. As a result, there is a growing need for specific tools capable of estimating the risks of toxicity and death to guide individualized therapeutic decisions, balancing treatment efficacy against tolerability.

To address these challenges, many oncologists have adopted an interdisciplinary approach that integrates principles from oncology and geriatrics to optimize care for older cancer patients. Consequently, many tools and assessments traditionally used by geriatricians are increasingly incorporated into routine oncologic and hematologic evaluations. The importance of baseline health status and well‐being at cancer diagnosis has been captured by PS, first quantified in the 1950s using the Karnofsky Performance Status (KPS) scale or the Eastern Cooperative Oncology Group (ECOG) scale to estimate a patient's ability to tolerate chemotherapy [28, 29].

Although PS remains a useful marker of a patient's clinical condition at diagnosis, frailty is a more complex, multidimensional, and dynamic syndrome. In recent years, frailty has been described in several cohorts of older patients with hematological malignancies. Frailty identifies a state of increased vulnerability to adverse outcomes, but there is substantial heterogeneity in how it is measured, with most studies employing a Geriatric Assessment (GA) to detect it [30].

### 1.3 Fitness in AML

#### 1.3.1 Ferrara Score

In 2013, an expert panel convened under the auspices of the Italian Society of Hematology (SIE), the Italian Society of Experimental Hematology (SIES), and the Italian Group for Bone Marrow Transplantation (GITMO) proposed consensus criteria to define “unfitness,” focusing on the planned therapeutic strategy [17]. Patients were classified as unfit for IC if they met at least one of nine criteria, including specific comorbidities, advanced age, low PS, and cognitive impairment. Similarly, unfitness for non‐IC was defined as at least one of six criteria that markedly worsen the clinical condition and preclude tolerance to even lower‐intensity regimens [17].

The application of these criteria showed high reliability in predicting outcomes after intensive treatment. Recently, Palmieri et al. retrospectively analyzed 655 adult AML patients treated with intensive regimens (standard “7 + 3,” CLAG‐M, or reduced‐dose CLAG‐M). Of these, 197 (30%) met at least one criterion for unfitness for IC (F‐unfit). Compared with patients without unfitness criteria (F‐fit), F‐unfit patients had significantly shorter overall survival (OS; median 4.8 vs. 36.8 months, *p* < 0.001) [31]. When compared with standard treatment‐related mortality (TRM) scores, the Ferrara unfitness assessment was more accurate in predicting 28‐day and 100‐day mortality. Its predictive performance could be further improved by including additional covariates such as PS and albumin levels. However, despite its precision in predicting early mortality, the Ferrara score was less accurate in predicting long‐term OS. This likely reflects the dominant impact of disease biology on relapse beyond the first 100 days, which ultimately determines OS [31].

Subsequently, these criteria were also validated in patients with secondary AML treated with CPX‐351. Palmieri et al. showed that patients classified as fit according to SIE/SIES/GITMO criteria had a significantly longer OS (median 18 vs. 8 months) and lower mortality at 28 days (4.6% vs. 10.7%) and 100 days (14.3% vs. 32%) compared to unfit patients [32]. A key limitation of the Ferrara criteria is their development in 2013, when the AML therapeutic landscape was very different. At that time, IC was the only curative option, while patients unfit for intensive treatment were restricted to HMA or low‐dose cytarabine (LDAC) as monotherapy. Those considered unfit even for non‐intensive regimens could receive only BSC [33].

Over the last decade, the AML scenario has changed profoundly with the introduction of several new agents. Venetoclax (VEN), in particular, has become the standard of care in combination with HMAs for treatment‐naïve (TN) AML patients ineligible for IC, following the VIALE‐A trial [3, 4, 6]. The advent of this combination and other therapeutic options for patients who are ineligible for IC highlights the need to distinguish between those who may benefit from these regimens and those who are unfit even for non‐intensive treatment. Although the Ferrara criteria were introduced before the advent of the HMA/VEN combination, these tools have also demonstrated prognostic value in patients treated with this regimen.

In a retrospective analysis of 103 patients treated with HMA/VEN for either TN or RR AML, OS did not differ significantly between patients classified as fit or unfit for IC according to the SIE/SIES/GITMO criteria. In contrast, patients considered unfit for non‐IC had significantly shorter OS in both the TN and RR cohorts. When patients were stratified into three groups—INT‐Fit, INT‐Unfit/NINT‐Fit, and NINT‐Unfit—the adverse prognostic impact of being NINT‐Unfit was confirmed in TN patients and showed borderline significance in the RR group [34].

Nowadays, being unfit for HMA/VEN is not necessarily associated with the impossibility of receiving treatment, but rather with the need for adapted therapeutic and supportive strategies. Patients considered unfit for standard‐dose HMA/VEN may still benefit from HMA monotherapy or from modified HMA/VEN schedules in which the duration of venetoclax exposure is reduced without compromising efficacy [35, 36].

#### 1.3.2 Hematopoietic Cell Transplantation‐Specific Comorbidity Index

The development of the Hematopoietic Cell Transplantation‐Specific Comorbidity Index (HCT‐CI) in 2005 enabled systematic incorporation of pre‐transplant comorbidities into the decision‐making [32]. The HCT‐CI aims to predict non‐relapse mortality (NRM) across hematological malignancies by assigning weighted points to individual comorbidities. In the original validation study, HCT‐CI scores of 0, 1, 2, 3, 4, 5, and ≥ 6 were associated with a stepwise increase in 2‐year NRM and a corresponding decrease in 2‐year survival [37, 38, 39]. The prognostic impact of comorbidities in AML was confirmed in a retrospective analysis, showing that a composite model comprising an augmented HCT‐CI, age, and cytogenetic/molecular risk had excellent predictive value in AML patients in first complete remission (CR1) undergoing transplantation [40]. Despite the absence of formal validation in AML patients treated with chemotherapy alone, the HCT‐CI is frequently applied in this setting as well. A major limitation of the index, however, is that it does not include age or disease‐specific biological parameters as markers of high toxicity risk. This suggests that models integrating cytogenetic and biological features, age, and HCT‐CI may provide accurate survival estimates in AML patients treated with either intensive or non‐intensive regimens.

Nevertheless, its role in the current AML landscape for elderly patients remains unclear. Data on its utility in non‐intensive treatment settings are scarce, and in the elderly population, activities of ADL, PS, frailty phenotype, and social fitness are at least as important as the presence of comorbidities. Because these dimensions are not captured by HCT‐CI, its use should be considered only in conjunction with additional parameters.

Comorbidities included in HCT‐CI are summarized in Table 1.

**TABLE 1:** Transplantation‐Specific Comorbidity Index (HCT‐CI): Definitions and weights of comorbidities.

Comorbidity	Definition (example/summary)	HCT‐CI weight
Mild pulmonary disease	FEV1 or DLCO 66%–80% predicted; or dyspnea on slight exertion; or moderate asthma	1
Moderate/severe pulmonary disease	FEV1 or DLCO ≤ 65% predicted; or dyspnea at rest; or oxygen dependence; severe COPD	2
Coronary artery disease	History of myocardial infarction, angina, or prior coronary revascularization	1
Congestive heart failure	Symptomatic heart failure or reduced left ventricular ejection fraction	2
Cardiac arrhythmia	Atrial fibrillation/flutter, sick sinus syndrome, or significant ventricular arrhythmias	1
Prior solid tumor	Any treated solid tumor (excluding non‐melanoma skin cancer), in remission	3
Mild hepatic disease	Chronic hepatitis or mild liver dysfunction without cirrhosis or portal hypertension	1
Moderate/severe hepatic disease	Cirrhosis, portal hypertension, or hepatic failure	3
Renal impairment	Serum creatinine > 2.0 mg/dL, dialysis, or history of renal transplantation	2
Diabetes mellitus	Diabetes requiring oral agents and/or insulin	1
Peptic ulcer disease	History of peptic ulcer requiring treatment or hospitalization	2
Inflammatory bowel disease	Crohn's disease or ulcerative colitis	1
Rheumatologic disease	Systemic lupus erythematosus, rheumatoid arthritis, or other connective tissue disease	2
Active infection	Infection requiring ongoing systemic therapy at time of assessment	1
Obesity	Body mass index (BMI) > 35 kg/m^2^	1
Cerebrovascular disease	History of stroke or transient ischemic attack	1
Psychiatric disturbance	Depression, anxiety disorder, or psychosis requiring medical treatment	1

*Note:* Total HCT‐CI score is the sum of all weights; higher scores indicate greater comorbidity burden and higher predicted non‐relapse mortality.

Abbreviations: BMI, body mass index; COPD, chronic obstructive pulmonary disease; DLCO, diffusing capacity of the lung for carbon monoxide; FEV1, forced expiratory volume in 1 s.

#### 1.3.3 Charlson Comorbidity Index

The aim of the Charlson Comorbidity Index (CCI) was originally developed to estimate the risk of treatment‐related toxicities and to predict outcomes in patients with multiple comorbidities. It incorporates 19 different medical conditions and has demonstrated prognostic value across a variety of underlying diseases, including solid and hematologic malignancies. The most commonly used version is the Deyo modification, which includes 17 variables: three of the original CCI categories (leukemia, lymphoma, and localized solid tumors) are combined into a single variable called “any malignancy other than metastatic solid tumors” [41, 42].

The prognostic role of the CCI in AML patients is not fully established. In a large retrospective analysis of 50 668 AML patients aged > 60 years, diagnosed and treated between 2004 and 2014, a higher CCI independently predicted worse early mortality and shorter OS. Higher CCI scores were more frequent with advancing age. Furthermore, patients with a higher CCI were less likely to receive chemotherapy or HSCT at a time when no curative alternatives to intensive treatment were available [43]. Conversely, other studies failed to demonstrate a significant association between higher CCI scores and survival in AML [44].

More recently, a 2023 study reported the first use of the CCI in AML patients deemed unfit for IC treated with HMA/VEN.

Investigators analyzed 100 TN patients finding that those with a CCI ≤ 6 had significantly longer OS than those with a CCI score of > 6 (8.8 vs. 3.4 months, *p* = 0.013). In the RR cohort, patients with a CCI < 4 had significantly better OS (9.4 vs. 5.2 months, *p* = 0.048).

A detailed comparison showed that, in the TN group, patients with CCI scores ≤ 6 were significantly younger, while the distribution of ELN 2022 categories and the use of HSCT did not differ. In RR groups, median age was again significantly lower in the ≤ 4 versus > 4 cohorts (54 vs. 70 years, *p* < 0.0001), whereas ECOG PS and ELN 2022 adverse‐risk disease were similarly distributed [45].

Comorbidities included in the CCI are summarized in Table 2.

**TABLE 2:** Deyo modification of the Charlson Comorbidity Index (CCI): Comorbidities and weights.

Comorbidity	Definition (example/summary)	CCI weight
Myocardial infarction	Prior myocardial infarction	1
Congestive heart failure	Clinical heart failure or documented left ventricular dysfunction	1
Peripheral vascular disease	Claudication, prior peripheral revascularization, or peripheral arterial disease	1
Cerebrovascular disease	Stroke or transient ischemic attack, with or without residual deficit	1
Dementia	Clinical diagnosis of dementia or significant cognitive impairment	1
Chronic pulmonary disease	COPD, chronic bronchitis, emphysema, or other chronic lung disease	1
Connective tissue disease	Rheumatoid arthritis, SLE, systemic sclerosis, polymyositis, and so on.	1
Peptic ulcer disease	History of gastric or duodenal ulcer	1
Mild liver disease	Chronic hepatitis or mild liver dysfunction without cirrhosis	1
Diabetes mellitus (uncomplicated)	Diabetes without documented end‐organ damage	1
Diabetes mellitus with end‐organ damage	Nephropathy, retinopathy, neuropathy, or other complications	2
Hemiplegia or paraplegia	Motor deficit due to stroke or spinal cord disease	2
Moderate or severe renal disease	Creatinine ≥ 3.0 mg/dL, dialysis, or renal transplantation	2
Any malignancy (non‐metastatic)	Solid tumor, leukemia, or lymphoma without distant metastases	2
Moderate or severe liver disease	Cirrhosis, portal hypertension, or hepatic failure	3
Metastatic solid tumor	Solid tumor with distant metastases	6
AIDS/HIV infection	HIV infection with or without AIDS‐defining conditions	6

*Note:* Total CCI score is the sum of all weights; higher scores indicate greater comorbidity burden and higher predicted mortality.

Abbreviations: COPD, chronic obstructive pulmonary disease; SLE, systemic lupus erythematosus.

#### 1.3.4 Comprehensive Geriatric Assessment

The CGA is a multidimensional tool widely used in both solid tumors and hematologic malignancies. It encompasses validated scales that evaluate core geriatric domains—comorbidities, functional status, nutritional status, social environment, polypharmacy, cognition, and mood—all of which are known predictors of chemotherapy toxicity and prognosis [21, 22] By integrating these domains, CGA allows more personalized treatment planning and the implementation of targeted geriatric interventions [46, 47].

Klepin et al. analyzed 74 TN AML patients, reporting that OS was shorter in those with impaired objectively measured physical function, assessed using the Short Physical Performance Battery (SPPB), and in those with impaired cognition, measured with the Modified Mini‐Mental State Examination (3MS) [13].

Similarly, Min et al. evaluated 105 TN AML patients treated with cytarabine and idarubicin. A GA was performed before chemotherapy and included physical function, cognition, depression, distress, nutrition, and social support. Impaired physical function by SPPB was associated with a higher incidence of grade 3–4 infections (*p* = 0.024) and renal failure (*p* = 0.013). Cognitive impairment by MMSE was associated with grade 3–4 infections (*p* = 0.044) and prolonged hospitalization (*p* = 0.005). Moreover, impaired physical function by SPBB and depressive symptoms by GDS predicted lower survival [48].

As treatment paradigm for older AML patients shift, with several non‐intensive options emerging as potential curative, it is crucial to evaluate the role of CGA in patients receiving non‐intensive regimens. One of the first studies in this setting analyzed 195 older adults (63 with myelodysplastic syndrome and 132 with AML) treated with IC (*n* = 75), low‐intensity therapy (*n* = 73), or supportive care alone (*n* = 47). In multivariable analyses adjusting for PS, cytogenetics, and bone marrow blast percentage, survival was poorer in patients with higher HCT‐CI scores, impaired ADL (Barthel Index), and self‐reported fatigue (per the European Organization for Research and Treatment of Cancer [EORTC] QLQ‐C30) [49].

More recently, a CGA was performed in all patients enrolled in the CALGB 361101 study, a companion study to the randomized phase 2 trial CALGB 11002. CALGB 11002 evaluated the addition of bortezomib to decitabine in adults aged ≥ 60 years deemed unfit for intensive therapy. About 96 (52%) of the 115 patients enrolled in CALGB 11002 were also participated in 361 101. In multivariate analyses, higher comorbidity burden (HCT‐CI > 3), worse cognition (Blessed Orientation‐Memory‐Concentration score > 4), and lower baseline global QOL (EORTC) were significantly associated with shorter OS after adjustment for Karnofsky PS, age, and treatment arm. Dependence in IADL and cognitive impairment were associated with higher 6‐month mortality [50]. A key strength of CGA is its multidimensional nature. Recently, it has been further refined by incorporating serum albumin. A Chinese study validated a new tool, the IACA index, which includes four risk factors: IADL scales (8 vs. 6–7 vs. ≤ 5), Advanced age (> 75 years), comorbidities (CCI ≥ 3), and hypoalbuminemia (< 3.4 g/dL). Patients were categorized into low‐ (0 points), intermediate‐ (1–2 points), and high‐risk (≥ 3 points) groups. As expected, relapse/progression‐related mortality increased across these groups (23.8%, 58.8%, and 100%, respectively), while the 2‐year OS probabilities declined (47.7%, 20.2%, and 0%, respectively) [51].

#### 1.3.5 Frailty Scales

Most of the tools discussed above focus on comorbidities and objectively measurable physical or mental parameters. However, frailty is a broader and more complex construct that also encompasses reduced activity, decreased strength, comorbidities, and a compromised social environment. Because frailty is not easily captured by a single objective measure, many attempts have been made to simplify and operationalize this context.

The Canadian Study of Health and Aging (CSHA) CFS is a simple, clinically applicable tool that classifies older individuals according to vulnerability and risk of death [25]. Initially developed to predict early mortality among elderly patients in the emergency department, it has since been extended to other settings, including oncology and hematology [52, 53].

In the AML context, Vigna et al. evaluated the CFS and found that highly frail patients had higher incidence of infections (72.7% vs. 36.8%, *p* = 0.02) and hospitalizations (57.6% vs. 21.1%, *p* = 0.011) compared with less frail patients [54]. In recent years, the CFS has been incorporated into a more comprehensive assessment tool, the Hematopoietic Cell Transplantation Frailty Scale (HCT‐FS). The HCT‐FS combines the CFS with IADL, Timed Up and Go (TUG), grip strength, self‐rated health, history of falls, and serum albumin and C‐reactive protein levels. It aims to classify HCT candidates as fit, pre‐frail, and frail [55]. In the validation study of 298 transplanted patients (2018–2020), 103 (34.6%) were fit, 148 (49.7%) pre‐frail, and 47 (15.8%) were frail at first consultation. Two‐year OS for fit, pre‐frail, and frail patients was 82.9%, 67.4%, and 48.3% (*p* < 0.001), respectively, and 5.4%, 19.2%, and 37.7% (*p* < 0.001), respectively. In patients < 60 years, the 2‐year OS and NRM were 88.4%, 69.3%, and 53.1% (*p* = 0.002), and 5.8%, 22.8%, and 34.8% (*p* = 0.005), respectively. In patients ≥ 60, the OS and NRM were 75.5%, 63.8%, and 41.4% (*p* = 0.006), and 4.9%, 16.4%, and 42.1% (*p* = 0.001), respectively. These data suggest that frailty assessment complements, rather than replaces, age in risk stratification [55].

The HCT‐FS was recently evaluated in younger AML patients undergoing HSCT. Patients were stratified as fit (≤ 1), pre‐frail (> 1 and < 5.5), or frail (≥ 5.5). One‐ and two‐year NRM were 2.9%, 14%, and 35.5%, and 4.4%, 15.1%, and 46.5% in fit, pre‐frail, and frail patients, respectively. Corresponding 1‐ and 2‐year OS rates were 91.4%, 78.2%, and 58.1%, and 86.8%, 69.3%, and 40.1%, respectively. The 1‐year cumulative incidence of relapse (CIR) was 15.8%, 14.2%, and 12.9% in fit, prefrail, and frail patients, respectively, with no significant differences between the groups. In contrast, relapse‐free survival (RFS) was progressively worse with higher frailty: 1‐ and 2‐year RFS were 81.3% and 73.1% in fit, 71.8% and 57.7% in prefrail, and 51.6% and 40.6% in frail patients. Similarly, the 1‐ and 2‐year GVHD‐free, relapse‐free survival (GRFS) were 59.6%, 55%, and 32.3%, and 51%, 39.4%, and 25.1%, in fit, prefrail, and frail patients, respectively [56].

Additionally, US hematologists recently evaluated the Veteran Affairs Frailty Index (VA‐FI), which measures frailty using a deficit‐accumulation approach. After a median follow‐up of 252.5 days, veterans with moderate‐to‐severely frail had significantly worse survival compared to mildly frail and non‐frail veterans, and increasing VA‐FI severity was directly associated with higher mortality [57, 58]. Another integration model was recently tested in Italy [22]. Investigators evaluated an integrated model in 197 patients at diagnosis using the G8‐score, the HCT‐CI, and the AML‐score for complete remission (CR). In univariate Cox models, all three tools were predictive of OS. However, in multivariate analysis, only G8 and AML score remained independently associated with OS, while the HCT‐CI lost significance. Interestingly, combining the scores significantly enhanced their predictive capacity performance [22].

### 1.4 Integration of Patient‐ and Disease‐Related Factors in Fitness Assessment

Nowadays, several therapeutic options are available for adult AML patients who are considered unfit for IC. Despite the strong prognostic power of many tools in predicting factors that influence short‐ and long‐term outcomes in patients treated with IC or HSCT, data on clinical parameters that predict tolerability and efficacy for less intensive treatments remain limited.

With the advent of numerous new agents and combinations and the growing number of unfit AML patients who are candidates for low‐intensity regimens, a CGA—balancing the risks and benefits of each specific intervention—is mandatory. This is particularly relevant because, with the current therapeutic armamentarium, different fitness levels may correspond to distinct therapeutic approaches [6].

Given the heterogeneity of AML among patients in terms of disease characteristics, biological profiles, personal circumstances, and preferences, no single clinical or biological parameter can provide sufficient information for optimal treatment selection. Instead, a multifaceted evaluation is required. Over recent years, several models combining patient‐ and disease‐related variables have been developed for daily clinical practice; however, most have been explored primarily in the context of intensive treatment to identify patients who should avoid IC or HSCT due to a high risk–benefit ratio.

Even within intensive care settings, despite the availability of multiple tools, there is no clear consensus on the optimal method for fitness assessment, and the concept of “fitness” remains only partially defined and difficult to quantify. Recently, an expert panel convened on behalf of the ELN specifically addressed these issues and attempted to clarify the definition and clinical application of fitness in AML [17].

#### 1.4.1 Measurable Patient Factors

The ELN panel first agreed that fitness r reflects a comprehensive evaluation incorporating age, PS, comorbidities, and functional capacity. Chronological age alone should not define fitness. As general life expectancy increases, many individuals reach advanced age in relatively good health, and there is often poor correspondence between chronological and biological age. Indeed, TRM among physically fit older patients may be comparable to that observed in younger cohorts. Consequently, patients aged > 70 or even > 75 years may still benefit from intensive treatment, but careful assessment is required, particularly with respect to PS. Although poor PS is more frequent in older patients, multivariate analyses suggest that PS is at least as important as age in predicting OS in AML [59, 60, 61, 62].

Several scales have been validated to measure PS in AML, most notably the ECOG PS and Karnofsky PS [28, 29]. Moreover, a geriatric evaluation that includes objective measures of physical functioning is essential to accurately estimate the residual functional capacity of older adults and to guide treatment intensity.

#### 1.4.2 Patient Self‐Reported Factors

The ELN panel also highlighted that PS contains a subjective component that is influenced by the patient's own perception of health, including self‐reported quality of life (QoL) and social support. In fact, patient‐reported physical functioning is significantly associated with OS, independent of physician‐assessed PS [63]. Therefore, combining physician‐assessed PS and patient‐reported outcomes may improve prognostic accuracy.

Social support is another parameter that is often underestimated. A meta‐analysis demonstrated that better perceived social support, stronger social networks, and being married were associated with improved OS in cancer patients, including those with leukemia and lymphoma [64]. A less strong social support negatively affects prognosis, being associated with higher rates of non‐adherence to treatment, shorter OS, and an increased likelihood of death or hospital readmission within 90 days of discharge [65, 66].

In light of these findings, QoL and social support should be explicitly integrated into the decision‐making process, with a primary goal of avoiding deterioration of the patient's baseline QoL. This is an endpoint increasingly incorporated into AML clinical trials. Studies in oncology (not specific to AML) have also shown that routine monitoring of patient‐reported symptoms is associated with longer survival compared with standard follow‐up, possibly because it promotes active patient engagement and facilitates earlier intervention [67, 68].

#### 1.4.3 Disease Factors

The ELN expert panel further emphasized that patient fitness coexists with “disease fitness.” It is well established that patients with unfavorable genetic risk have lower remission rates and shorter OS when treated with IC, irrespective of age or PS [6]. There is growing interest in identifying biological profiles that respond preferentially to specific therapies including IC, CPX‐351, HMA/VEN combinations, and targeted agents. For example, in a small propensity‐matched cohort of AML patients aged > 65 years, ELN adverse risk disease and RUNX1 mutations were associated with superior OS with VEN‐AZA, whereas IC was superior in patients with ELN intermediate‐risk disease [69]. Conversely, other studies have reported no significant differences in early mortality or OS between patients treated with CPX‐351 or VEN‐AZA, even in high‐risk AML [70, 71].

Recently, the ELN 2024 recommendations stratified AML patients eligible for HMA/VEN into three risk categories based on outcomes, underscoring the observation that specific biological profiles show greater sensitivity to certain agents [72]. From this perspective, a complete biological and cytogenetic work‐up is mandatory even in patients who are not candidates for intensive treatment. For example, in patients with IDH1 mutations, the combination of ivosidenib and azacitidine has demonstrated excellent efficacy, a favorable safety profile, and durable responses [73]. Similarly, FLT3‐mutated disease is now targetable with gilteritinib at relapse, and several studies are exploring the integration of FLT3 inhibitors into first‐line therapy [74, 75, 76, 77].

Many additional oral, targeted, small‐molecule inhibitors are currently in clinical development. Randomized trials are ongoing to compare standard cytotoxic chemotherapy (e.g., “7 + 3” or CPX‐351) versus lower‐intensity VEN‐based regimens in older, fit patients who are candidates for allo‐SCT. An approach based on early disease control with either strategy, followed by timely HSCT, may result in more durable remissions [78].

**FIGURE 1:**
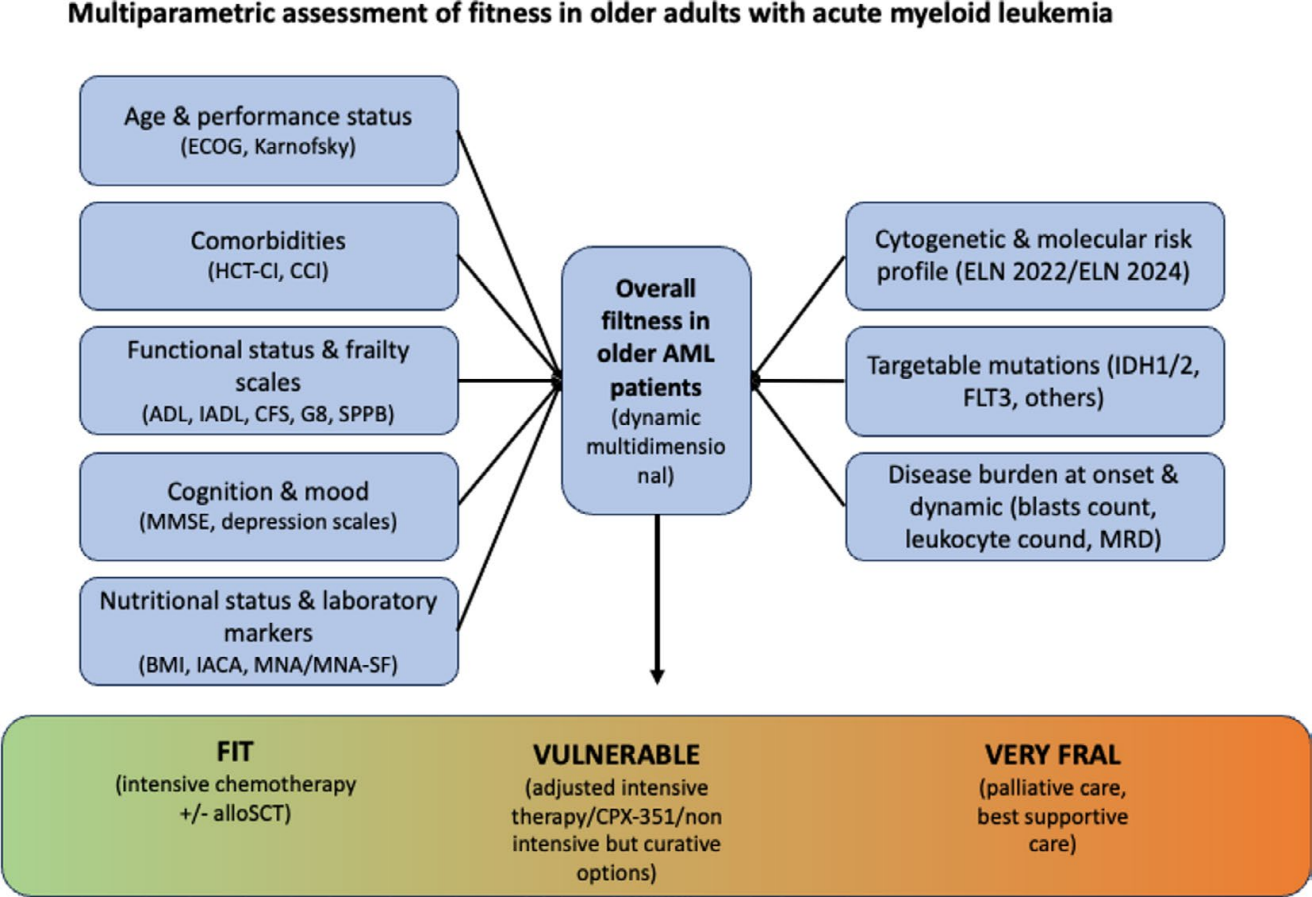
Multiparametric assessment of fitness in older adults with acute myeloid leukemia. Fitness is conceptualized as a dynamic, multidimensional construct resulting from the integration of patient‐related and disease‐related domains. Patient‐related factors include age and PS, comorbidities (e.g., HCT‐CI, CCI), functional status and frailty (ADL, IADL, SPPB, G8, CFS, HCT‐FS, VA‐FI), cognition and mood, nutritional status and laboratory markers (e.g., albumin, IACA index), and QoL and social support. Disease‐related factors include cytogenetic and molecular risk (ELN 2022/2024), targetable mutations (e.g., IDH1/2, FLT3), disease burden and dynamics, and transplant eligibility. The integrated assessment of these domains informs the selection of therapeutic strategies along a continuum of intensity, ranging from IC with or without allogeneic transplantation in fit patients to non‐intensive combinations or palliative care in frail and very frail patients.

A schematic representation of the proposed multiparametric assessment of fitness, integrating patient‐related and disease‐related domains, is shown in Figure 1, while the specific thresholds and clinical impact of each domain are summarized in Table 3. Based on the integrated assessment of these domains, patients can be categorized into four fitness levels—fit, vulnerable, unfit, and very frail—each associated with different therapeutic strategies (Table 4).

**TABLE 3:** Proposed multiparametric clinical assessment: Domains, thresholds for unfit/frail status, and clinical impact.

Domain	Specific parameter	Threshold (unfit/frail)	Clinical impact (from literature)
Performance	ECOG PS/Karnofsky PS	ECOG > 2 or Karnofsky < 70%	Strong predictor of reduced overall survival, at least as important as age [28, 29].
Comorbidities	HCT‐CI score/CCI	HCT‐CI > 3 and/or CCI > 6	Associated with higher non‐relapse mortality and reduced overall survival [32, 37, 38, 39, 40, 41, 42, 43, 44, 45].
Physical functioning	SPPB (gait speed component)	Reduced gait speed	Increased risk of grade 3–4 infections and renal impairment [48].
Cognitive status	MMSE/3MS	Impaired cognitive performance	Associated with prolonged hospitalization and lower adherence to treatment [48].
Nutritional status	Serum albumin	< 3.4 g/dL	Component of the IACA index; associated with reduced overall survival [51].
Social support	Living status/social network	Living alone or limited social support	Associated with reduced overall survival and higher risk of unplanned readmission [64, 65, 66].
Disease features	ELN 2022/ELN 2024 risk group; key mutations	Adverse‐risk category; TP53 mutation	Predicts treatment resistance and poor outcome, independently of performance status [6, 72].

*Note:* This keeps exactly your domains and “Valore di Soglia (Unfit/Frail)” and “Impatto Clinico,” only standardized in English and with abbreviations explained.

Abbreviations: 3MS, Modified Mini‐Mental State Examination; CCI, Charlson Comorbidity Index; ECOG PS, Eastern Cooperative Oncology Group performance status; ELN, European LeukemiaNet; HCT‐CI, Hematopoietic Cell Transplantation‐Specific Comorbidity Index; IACA, IADL–Age–CCI–Albumin index; MMSE, Mini‐Mental State Examination; SPPB, short physical performance battery.

**TABLE 4:** Suggested therapeutic strategy according to fitness level in older adults with AML.

Fitness level	Clinical profile	Suggested therapeutic strategy
1. Fit	ECOG 0–1; HCT‐CI < 2; preserved cognition; adequate social support	Intensive chemotherapy ± alloSCT. Prioritize performance status over chronological age when considering intensive regimens and transplant.
2. Vulnerable (intermediate fit)	ECOG 1–2; 1–2 relevant comorbidities; mild impairment of gait speed (SPPB); generally preserved cognition and social support	Intensive chemotherapy with dose or schedule adjustments; CPX‐351 in secondary AML; or reduced‐dose standard chemotherapy; or HMA/venetoclax. Always integrate biological risk and targetable mutations.
3. Unfit (frail)	At least one SIE/SIES/GITMO criterion defining unfitness for intensive chemotherapy; CCI > 6; often living alone or limited social support	Non‐intensive combination therapy, with HMA/venetoclax as current standard of care. Consider disease biology (e.g., ELN risk, targetable mutations) when selecting specific non‐intensive regimens.
4. Very frail (non‐intensive unfit)	ECOG ≥ 2 and/or Karnofsky < 70%; at least one SIE/SIES/GITMO criterion defining unfitness for non‐intensive therapy; cognitive impairment; serum albumin < 3.0 g/dL	Predominantly palliative approach/best supportive care. Hydroxyurea, LDAC, or oral decitabine may be used for symptom control only. Primary goal: maintenance or improvement of quality of life.

Abbreviations: alloSCT, allogeneic stem cell transplantation; CCI, Charlson Comorbidity Index; ECOG, Eastern Cooperative Oncology Group; GITMO, Gruppo Italiano Trapianto di Midollo Osseo; HCT‐CI, Hematopoietic Cell Transplantation‐Specific Comorbidity Index; HMA, hypomethylating agents; LDAC, low‐dose cytarabine; SIE, Società Italiana di Ematologia; SIES, Società Italiana di Ematologia Sperimentale; SPPB, short physical performance battery.

## 2 Conclusions

Fitness should be viewed as a dynamic parameter rather than a static classification. Patients may initially be categorized as unfit for intensive treatment due to organ dysfunction or poor PS related to the leukemia itself. In such cases, an initial non‐intensive approach may limit toxicity while preserving the possibility of subsequent high‐intensity consolidation, including allo‐HSCT, if PS and organ function improve after achieving remission. Conversely, patients classified as fit at diagnosis may become frail due to treatment‐related toxicities or disease progression.

In the contemporary AML therapeutic landscape, fitness assessment is becoming increasingly complex but also more crucial. Objective and subjective patient factors—including age, PS, comorbidities, functional status, cognition, QoL, and social support—must be integrated with disease‐related features such as cytogenetic features, molecular abnormalities, and anticipated sensitivity to specific agents. In this evolving setting, the development of advanced decision‐support tools is of particular interest. The advent of artificial intelligence (AI) and machine learning may facilitate the creation of integrated prognostic models that combine clinical, geriatric, and biological variables to refine risk stratification and guide the selection and timing of intensive, non‐intensive, and targeted therapies. In recent years, increasing attention has been directed toward the application of machine learning in AML, with diverse applications facilitating rapid and accurate diagnosis, refined risk stratification, and optimization of therapeutic strategies [79]. Such approaches have the potential to improve individualized treatment strategies and optimize outcomes for older adults with AML.

## Author Contributions

**Antonella Bruzzese**, **Enrica Antonia Martino**, **Fortunato Morabito**, **Ernesto Vigna**, and **Massimo Gentile:** conceptualization. **Antonella Bruzzese**, **Enrica Antonia Martino**, **Francesco Mendicino**, **Ernesto Vigna**, and **Fortunato Morabito:** methodology. **Enrica Antonia Martino**, **Santino Caserta**, **Fortunato Morabito**, **Massimo Gentile**, and **Nicola Amodio:** writing – original draft preparation. **Antonella Bruzzese**, **Enrica Antonia Martino**, **Santino Caserta**, **Fortunato Morabito, Ernesto Vigna**, and **Massimo Gentile:** writing – review and editing. All authors have read and agreed to the published version of the manuscript. All authors contributed equally to the preparation of this manuscript.

## Funding

The authors have nothing to report.

## Conflicts of Interest

The authors declare no conflicts of interest.

## Data Availability

The authors have nothing to report.
